# *RICE CENTRORADIALIS 1*, a *TFL1*-like Gene, Responses to Drought Stress and Regulates Rice Flowering Transition

**DOI:** 10.1186/s12284-020-00430-3

**Published:** 2020-09-24

**Authors:** Yan Wang, Yuyang Lu, Ziyu Guo, Yanfeng Ding, Chengqiang Ding

**Affiliations:** 1 College of Agriculture, Nanjing Agricultural University, Nanjing, 210095 People’s Republic of China; 2 College of Biology and Environmental Sciences, Jishou University, Jishou, 416000 People’s Republic of China; 3 Key Laboratory of Crop Physiology Ecology and Production Management, Nanjing Agricultural University, Nanjing, 210095 People’s Republic of China; 4 Jiangsu Collaborative Innovation Center for Modern Crop Production, Nanjing Agricultural University, Nanjing, 210095 People’s Republic of China

**Keywords:** Drought, Florigen, Flowering transition, Heading time, *RCN1*, Rice

## Abstract

**Background:**

The initiation of flowering transition in rice (*Oryza sativa*) is a complex process regulated by genes and environment. In particular, drought can interfere with flowering; therefore, many plants hasten this process to shorten their life cycle under water scarcity, and this is known as drought-escape response. However, rice has other strategies; for example, drought stress can delay flowering instead of accelerating it. *RICE CENTRORADIALIS 1* (*RCN1*) is a *TERMINAL FLOWER*-like gene that influences rice flowering transition and spike differentiation. It interacts with 14–3-3 proteins and transcription factor OsFD1 to form a florigen repression complex that suppresses flowering transition in rice.

**Results:**

In this study, we explored the role of *RCN1* in the molecular pathway of drought-regulated flowering transition. The *rcn1* mutant plants displayed early heading under both normal water and drought stress conditions, and they were more insensitive to drought stress than the wild-type plants. Abscisic acid (ABA) signaling-mediated drought-induced *RCN1* is involved in this process.

**Conclusions:**

Thus, RCN1 plays an important role in the process of drought stress inhibiting flowering transition. It may worked by suppressing the protein function rather than transcription of *HEADING DATE 3a*.

## Background

Drought is one of the most severe natural stresses affecting crops. Owing to inadequate irrigation plans, at least 30% of the total rice cultivation area worldwide is rainfed (Dixit et al. 2014). Because water resources are unevenly distributed in terms of both space and time, rice fields are severely affected by uneven rainfall, which leads to seasonal water shortages (Wu et al. 2011). Rice, which is consumed by more than half of the global population, has been grown under flooded irrigation conditions over the course of its evolution as a food crop because it requires large quantities of water and is sensitive to water-deficient soils. Approximately 3000–5000 L of water is used to produce 1 kg of rice, and this is almost twice the amount needed to grow other crops such as maize and wheat (Dixit et al. 2014). Drought can lead to a considerable delay in rice plant growth and spikelet development, thereby resulting in low crop yield, which causes substantial economic losses. Thus, understanding the molecular mechanisms underlying the effects of drought on key developmental processes in rice is crucial for the production and breeding selection of this crop.

Floral transition marks the switch from vegetative growth to reproductive growth. The timing of floral transition is a key determinant of crop production and adaptability to complex environmental conditions. The effects of drought on flowering time have been documented in several plant species (Bernal et al. 2011; Bocco et al. 2012; Franks 2011; Riboni et al. 2013; Riboni et al. 2016; Sherrard and Maherali 2006). One strategy of plants to endure water scarcity is to accelerate flowering transition, and this is known as drought-escape (DE) response. This response enables plants to accelerate the completion of their life cycle and produce seeds before the stress conditions become too severe (Franks 2011; Riboni et al. 2013; Riboni et al. 2016). The molecular mechanisms underlying the regulation of flowering time under drought stress have recently been elucidated in *Arabidopsis*. In this species, the positive regulator *GIGANTEA* (*GI*) and the florigen genes *FLOWERING LOCUS T* (*FT*) and *TWIN SISTER OF FT* (*TSF*) play key roles in regulating the DE response, which occurs only under long-day (LD) growth conditions. Abscisic acid (ABA)-deficient mutants, *aba1* and *aba2*, flowered later than wild type (WT) plants and showed a reduced DE response under LD conditions (Riboni et al. 2016), implying that drought stress positively regulates the transcription of these florigens in an ABA-dependent manner (Riboni et al. 2013; Riboni et al. 2016).

In addition, ABA has been found to repress flowering independent of florigen genes. The flowering-promoting gene SUPPRESSOR OF OVEREXPRESSION OF CONSTANS1 (*SOC1*) was repressed by ABA in the shoot apical meristem (Riboni et al. 2016). Rice plants delay flowering under drought stress to avoid reproductive growth under adverse conditions (Bocco et al. 2012; Fukai et al. 1999; Galbiati et al. 2016; Zhang et al. 2006). Drought stress strongly reduces the transcription of EARLY HEADING DATE 1 (*Ehd1*), which is upstream of florigen genes, and accumulation of HEADING DATE 3a (*Hd3a*) and RICE FLOWERING LOCUS T1 (*RFT1*), and ultimately leads to delayed flowering (Galbiati et al. 2016). However, a recent study reported that rice responds to drought differently, depending on its intensity, suggesting that a less severe drought condition could trigger the DE response (Du et al. 2018). The drought response of rice depends on the ABA-signaling pathway, similar to that of *Arabidopsis*. Furthermore, the regulation of rice flowering under drought conditions is not dependent on the photoperiod, unlike that of *Arabidopsis* (Zhang et al. 2016). *Early heading date 1* (*Ehd1*) is specific for flowering in rice (Doi et al. 2004), and it plays a key role in integration of drought stress and the photoperiodic signals (Galbiati et al. 2016). *Grain number, plant height, and heading date 7* (*Ghd7*) is another specific gene in rice, which involved in the regulation of DE (Du et al. 2018; Wu et al. 2008). *OsGIGANTEA* (*OsGI*) does not integrate photoperiodic and drought signals as in *Arabidopsis* (Galbiati et al. 2016). Indicating that the molecular mechanism underlying flowering initiation differs between these two species.

Florigen is the ultimate regulator of flowering under varied environmental conditions. It belongs to the family of phosphatidylethanolamine-binding proteins (PEBPs). TERMINAL FLOWER 1 (TFL1), an anti-florigen, which also belongs to this family, suppresses flowering and thus opposes the actions of FT in mediating flowering transition, although they are expressed in a similar pattern and have similar structures (Guo et al. 2014; Hanano and Goto 2011). RICE CENTRORADIALIS 1 (*RCN1*), a rice *TFL1* homolog, performs functions similar to those of *TFL1* in the flowering regulation process (Nakagawa et al. 2002; Zhang et al. 2005). A genome-wide association study of a population of 950 rice varieties collected worldwide identified *RCN1* as an important locus associated with flowering time (Huang et al. 2012). The underlying molecular mechanism of *RCN1* has been studied recently. The RCN1 protein is synthesized in the leaves and transported to the shoot apical meristem (SAM), where it opposes the action of florigen in floral transitions by competing with it to form complexes with the 14–3-3 and OsFD1 proteins (Kaneko-Suzuki et al. 2018). In addition, the genome-wide association study found that *RCN1*, located within one quantitative trait locus (q12), is related to drought tolerance during the vegetative phase (Hoang et al. 2019). Nevertheless, the roles of *RCN1* in the integration of environmental signals in rice are poorly understood.

In the present study, the mutation of *RCN1* significantly advanced the heading date of rice. Moreover, we suggest an ABA-dependent response pathway for drought stress-induced *RCN1* expression. In this pathway, two transcription factors, *ABA-RESPONSIVE ELEMENT BINDING PROTEIN 1* (*OsAREB1*) and *ABA RESPONSIVE ELEMENT (ABRE)-BINDING FACTOR* (*OSBZ8*)*,* are induced within minutes upon exposure to ABA and drought stresses and both function as *trans*-activators that modulate the drought response of *RCN1*.

## Results

### Influence of Drought on the Heading Date Is Weaker in *rcn1* Mutants than in WT Plants

To test if *RCN1* loss of function affects rice flowering time, we obtained two different insert mutations in the coding region of *RCN1* using the clustered regularly interspaced short palindromic repeats-associated protein-9 (CRISPR-Cas9) technology (Figure S1), the frame-shift mutations caused premature termination of translation or amino acid substitutions. In both mutant lines *RCN1* was not functional. The experiment was carried out during the growing season. Rice plants were subject to severe drought stress, as the soil relative water capacity (SRWC) was maintained between 30%–50% (Figure S2). In *rcn1* mutants, heading occurred approximately 7 days earlier than that in the WT plants under normal water conditions (Table 1), indicating a negative role of *RCN1* in controlling flowering transition. Both WT and *rcn1* mutant plants showed a significantly late-heading phenotype under severe drought conditions (Table 1). It is noteworthy that the heading date of the WT plants was delayed for approximately 14 days, and the two *rcn1* mutant plants showed delays of 9.2 and 10.9 days, respectively. The results indicated that *RCN1* loss of function reduced the effect of drought on rice heading date (Table 1).

**Table 1:** Heading date of rice plants (*n* = 15 per group per treatment) under normal water and drought stress conditions

	Normal water	Drought	Delayed days
Wild type	83.2 ± 4.3 b	97.4 ± 5 a	14.2
*rcn1–4*	72.8 ± 3.1 c	83.7 ± 4.1 b	10.9
*rcn1–11*	75.5 ± 2.8 c	84.7 ± 3.3 b	9.2

Different letters indicate statistically significant differences (*P* < 0.05, Duncan multiple-range test)

The expression of *RCN1* in the *rcn1* mutant plants was not significantly different from that of the wild-type plants under normal watering conditions (Figure S3a). In contrast, in *rcn1* mutant plants, *RCN1* showed a significantly decreased responsiveness to drought. The rice florigens *Hd3a* and *RFT1* promote flowering. Under normal watering conditions, there was no obvious difference in *Hd3a/RFT1* expression levels between *rcn1* mutants and wild-type plants. Under drought stress conditions, both the wild-type and the *rcn1* mutant plants showed a significant down-regulation of *Hd3a* and *RFT1*. However, *Hd3a* and *RFT1* were not regulated by *RCN1*; they were down-regulated under drought conditions in an *RCN1*-independent manner (Figure S3b, c). Drought stress can delay flowering by inhibiting florigen expression, and there was no effect of RCN1 on florigens transcription.

### Tissue-Specific Expression and Localization of RCN1

**Fig. 1:**
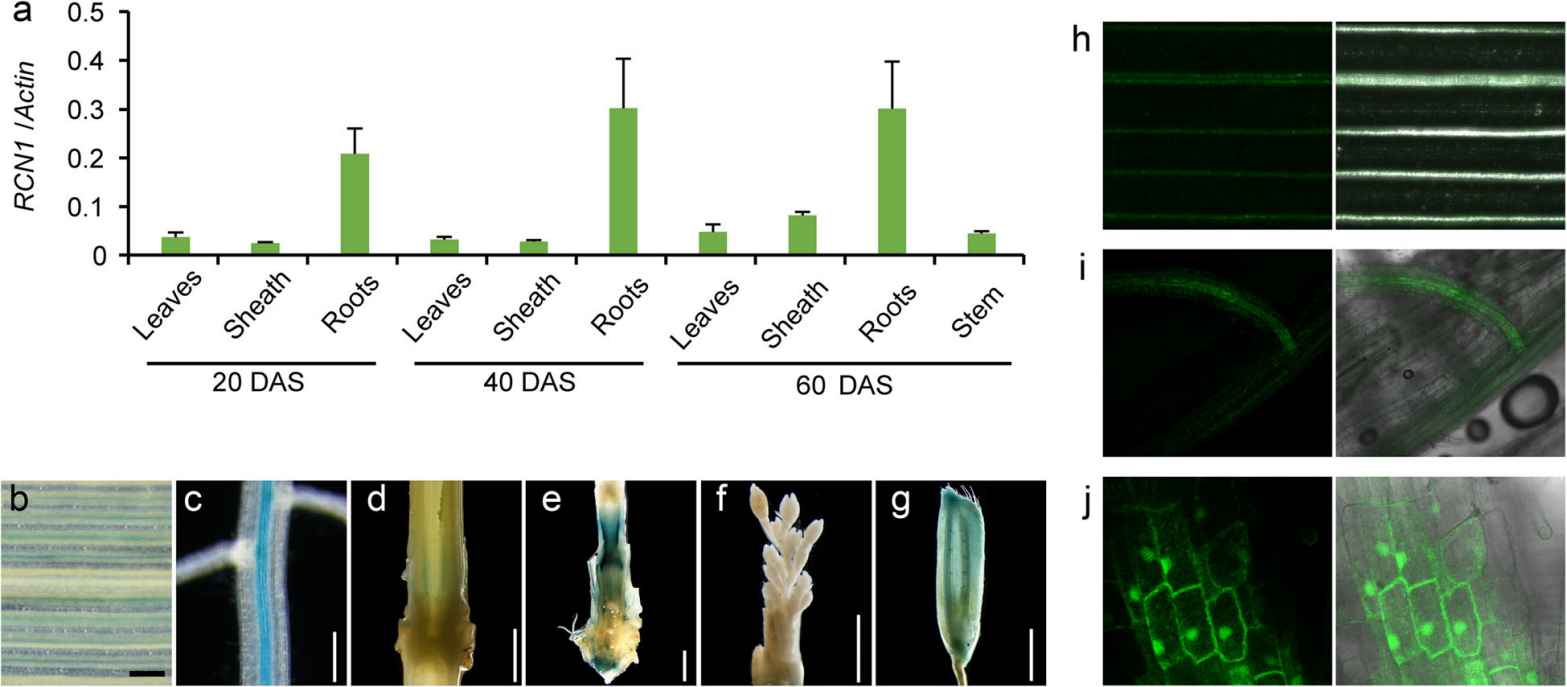
Expression patterns of *RCN1* in rice. Expression of *RCN1* RNA in rice (**a**). *RCN1* expression was analyzed by quantitative real-time PCR throughout the growth period. *Actin* RNA was used as the control. DAS: days after seeding. GUS staining images of *pRCN1::GUS:NOS* in transgenic rice leaf (**b**), root (**c**), stem node (**d**), basal internodes (**e**), young panicle (**f**), and glume (**g**). The localization of the RCN1-GFP protein was visualized in confocal images of the leaf (**h**) and root (**i**) in *pRCN1::RCN1-GFP:NOS* transgenic rice plants, and the subcellular localization of the RCN1-GFP protein was visualized lateral root cells (**j**). Bars = 100 μm (for **b**-**c**), 1 mm (for **d**-**g**)

To examine the spatio–temporal expression of *RCN1*, a quantitative reverse transcription- PCR (qRT-PCR) analysis was performed. The expression of *RCN1* was detected in the leaves, roots, and stems throughout the growth period, and the highest expression was observed in the roots (Fig. 1a). To further examine the expression pattern of *RCN1*, the 5′ distal region (4 kb) of the *RCN1* locus was fused with the beta-glucuronidase (GUS) reporter and used to conduct histochemical staining of the *GUS* gene. The expression of *GUS* was detected in the vascular tissues of the leaves and roots (Fig. 1b, c). In the cross-section of stems, a high *GUS* expression was detected in the nodes and basal internodes (Fig. 1d, e), whereas no expression was detected in young panicles (Fig. 1f). These results were consistent with the qRT-PCR results. Furthermore, *GUS* expression was observed in the glumes, and this has never been reported (Fig. 1g).

To examine the localization of the RCN1 protein in the plant, we constructed an expression vector with GREEN FLUORESCENT PROTEIN (*GFP*) as the reporter gene, by fusing the *RCN1*-coding region with the *GFP*-coding region (*RCN1-GFP*), and used the same promoter as that in the *GUS* analysis. The distribution of RCN1-GFP was consistent with the results of the qRT-PCR and *GUS* staining, suggesting the localization of RCN1 to the vasculature of the leaves and roots (Fig. 1h, i). We also observed the subcellular localization of RCN1-GFP in lateral root cells, where it was mainly distributed in the cell nucleus, cytoplasm, and cellular membrane (Fig. 1j); the same was observed in rice protoplasts (Figure S4).

### Drought Stress Activates *RCN1* Transcription in an ABA-Dependent Manner

**Fig. 2:**
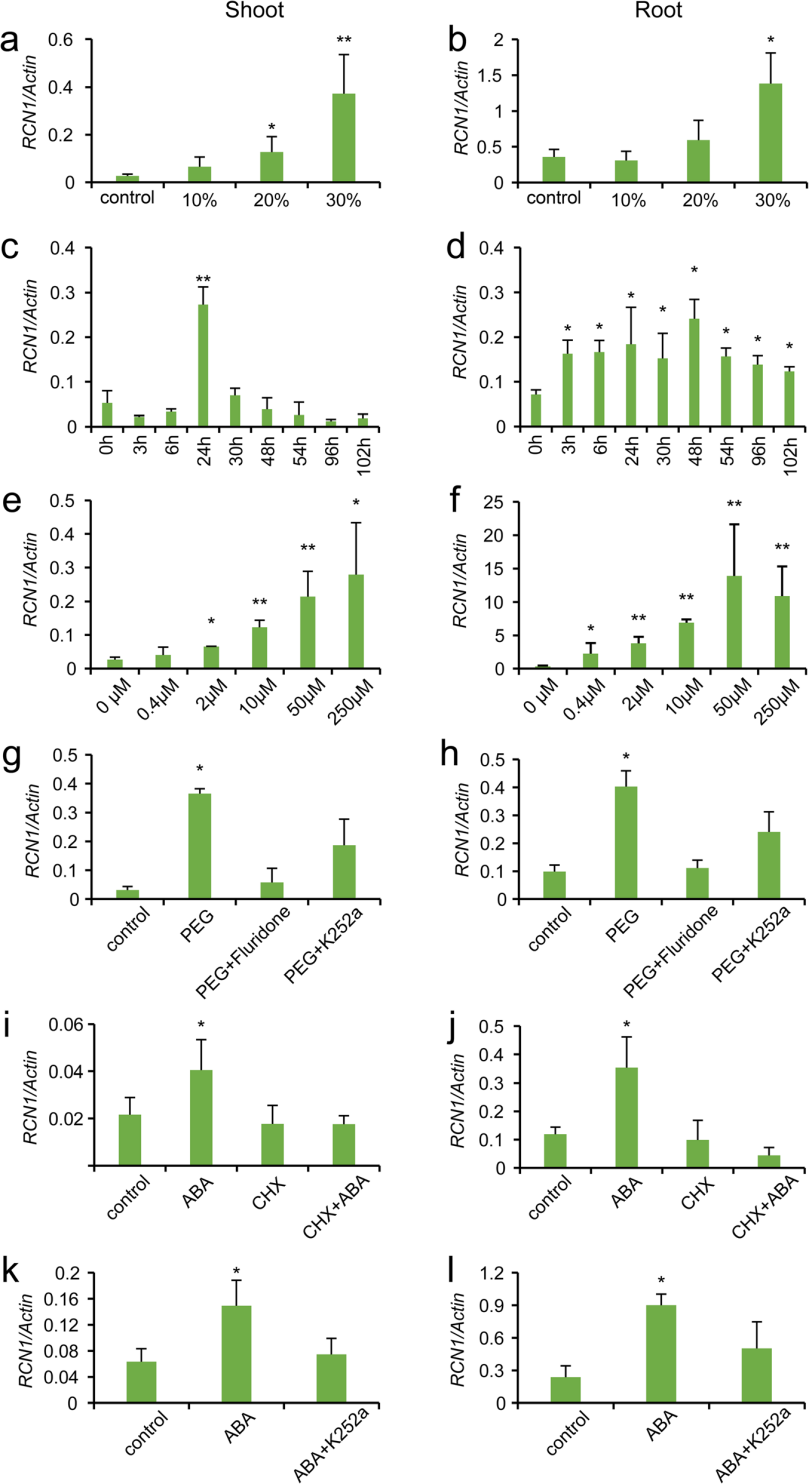
Expression levels of *RCN1* in the leaves and roots under different PEG6000 treatment concentrations (**a** and **b**), different ABA treatment times (**c** and **d**), and ABA treatment concentrations (**e** and **f**). PEG6000-induced *RCN1* levels in the leaves (**g**) and roots (**h**) depended on ABA synthesis and protein phosphorylation. ABA-induced *RCN1* levels in the leaf (**i**) and root (**j**) depended on de novo protein synthesis. CHX: cycloheximide. ABA-induced *RCN1* expression levels in the leaf (**k**) and (**l**) root depended on protein phosphorylation. Note: Statistical analysis was performed using the Student’s *t* test; significantly different values at *P* < 0.05 (*) or *P* < 0.01 (**) are indicated

For the osmotic stress treatment, plants were transferred to pots containing three different concentrations of polyethylene glycol (PEG6000), while those grown without PEG6000 were used as the controls. The expression of *RCN1* was detected by qRT-PCR. The results indicated that 30% PEG6000 strongly increased the expression of *RCN1* in the shoots and roots (Fig. 2a, b), and therefore this concentration was used in the subsequent experiments. It is widely known that ABA is accumulated in response to drought; thus, experiments were designed to verify whether *RCN1* would respond to ABA. We added 50 μM ABA to the rice culture solution and detected the expression level of *RCN1* at 0, 3, 6, 24, 30, 48, 54, 96, and 102 h of incubation, by qRT-PCR. Overall, ABA strongly increased the expression of *RCN1* in the shoots and roots, particularly in the latter (Fig. 2c, d). Because the upregulation of *RCN1* in the shoots was only detected 24 h post-ABA treatment, samples collected at this time point were used to evaluate the effects of ABA treatment. To evaluate the relationship between ABA and *RCN1*, we further examined the effect of different concentrations of exogenous ABA on *RCN1* expression. The results showed that *RCN1* expression was proportional to the exogenous ABA concentration applied (Fig. 2e, f). Compared with the control, the expression of *RCN1* in the shoots increased by up to 10-fold (Fig. 2e), far below the 39-fold observed in the roots (Fig. 2f). To identify if PEG6000 induced *RCN1* expression in an ABA-dependent manner, we treated rice plants with 40 μΜ fluridone for 30 min, and then moved them to 30% PEG6000 solution with fluridone. Fluridone, an inhibitor of carotenoids, which are the main precursors of ABA in plants (Raikhel et al. 1986; Kowalczyk-Schroder and Sandmann 1992), also inhibits the biosynthesis of ABA. Fluridone treatment was effective inhibit the increase in ABA content (Hsu et al. 2006; HSU et al. 2003; Perales et al. 2005; Shi et al. 2012). The results revealed that, after blocking ABA synthesis, *RCN1* did not respond to PEG6000 (Fig. 2g, h), suggesting that the PEG6000-mediated induction of *RCN1* was dependent on ABA. The ABA-mediated induction of *RCN1* was completely inhibited by cycloheximide (CHX), which is an inhibitor of protein synthesis (Fig. 2i, j). This indicated that protein synthesis is necessary to ABA regulate *RCN1* expression, one reason for the requirement may have been that transcription factors play an important role in regulation. PEG6000- and ABA-induced *RCN1* expression was partially restrained by K252a, which is a phosphorylation blocker (Fig. 2g, h, k, i). These results revealed an ABA-mediated induction of *RCN1* that was dependent on phosphorylation, and provided further evidence for ABA-mediated induction of *RCN1* under drought.

### ABA Induces *RCN1* through Transcription Factors

The basic leucine-zipper (*bZIP*) transcription factors are bound to the ABA-responsive *cis*-element *ABRE* in the promoters of ABA-inducible genes to regulate plant stress responses. The conserved sequence of *ABRE* is ACGT, and the C/G/A nucleotides flanking the ACGT core enhance bZIP protein binding specificity and affinity (Hattori et al. 2002). We screened 26 ABA response transcription factors from authoritative databases and selected five transcription factors that accumulated in response to ABA faster than *RCN1* (Figure S5a). A new ABA treatment experiment was then carried out to determine the expression levels of these transcription factors. Finally, *OsAREB1, OSBZ8,* and *TRAB1* showed a rapid and strong response to ABA in both leaves and roots (Figure S5b, c). Because the induction of *OsAREB1*, *OSBZ8*, and *TRAB1* expression by ABA was not blocked by CHX (Figure S5d, e), they appear to be primary ABA response genes.

**Fig. 3:**
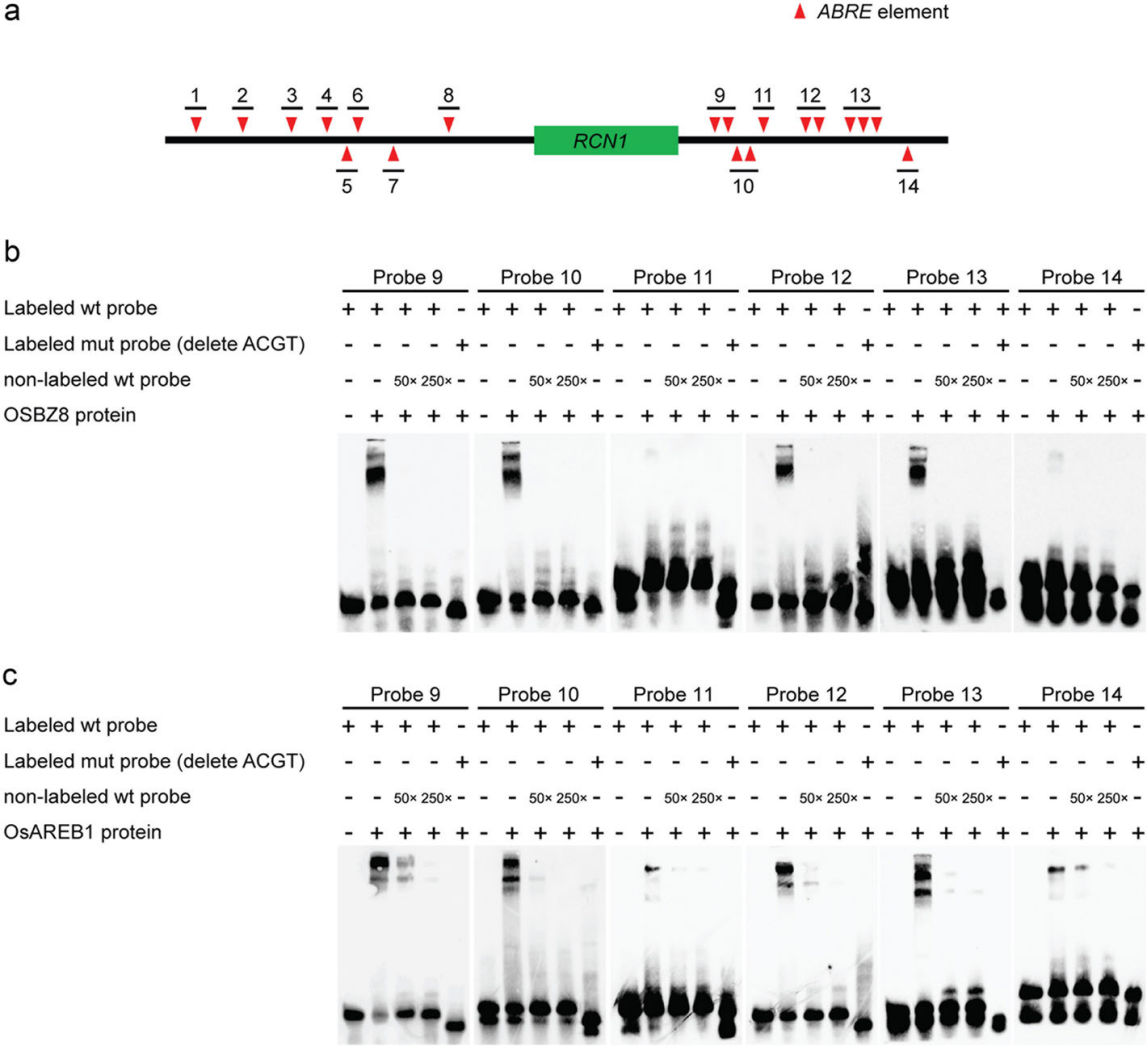
Location of the probes in the genomic DNA of *RCN1* (**a**). OSBZ8 (**b**) and OsAREB1 (**c**) were bound to the downstream sequence of *RCN1*. Note: 50×: 50 times concentration; 200×: 200 times concentration

Electrophoretic mobility shift assays (EMSA) were used to examine the binding activities of the OsAREB1, OSBZ8, and TRAB1 proteins with the *ABRE* element in the *RCN1* promoter. The 14 sequence specific fragments around the *RCN1* locus contained 19 ACGT elements that were synthesized and labeled with biotin (Fig. 3a). OsAREB1, OSBZ8, and TRAB1 were expressed and extracted from the BL21 competent *E. coli* strain. Among the six different fragments tested, OsAREB1 and OSBZ8 exhibited the strongest interaction with Probe 13 (containing three ACGT elements), a slightly weaker interaction with Probes 9, 10, and 12 (each containing two ACGT elements), and the weakest interactions with Probes 11 and 14 (each containing one ACGT element) (Fig. 3b, c). To examine the specificity of these interactions, a 50- or 200-fold unlabeled probe was applied to compete with the labeled probe. The interactions of OsAREB1 and OSBZ8 and labeled probes were inhibited by excess unlabeled probes. No interaction was detected with mutant probes that lacked the ABRE element sequence (Fig. 3b, c). There were no interactions between OsAREB1 and OSBZ8 and Probes 1–8 (Figure S6a, b), and between the TRAB1 protein and any of the 14 probes (Figure S6c). Therefore, OsAREB1 and OSBZ8 seemed to bind only to the *ABRE* element, which is located in the 3′ distal region of the *RCN1* locus, and their binding ability is likely be related to the number of *ABRE* elements included in the DNA fragments.

**Fig. 4:**
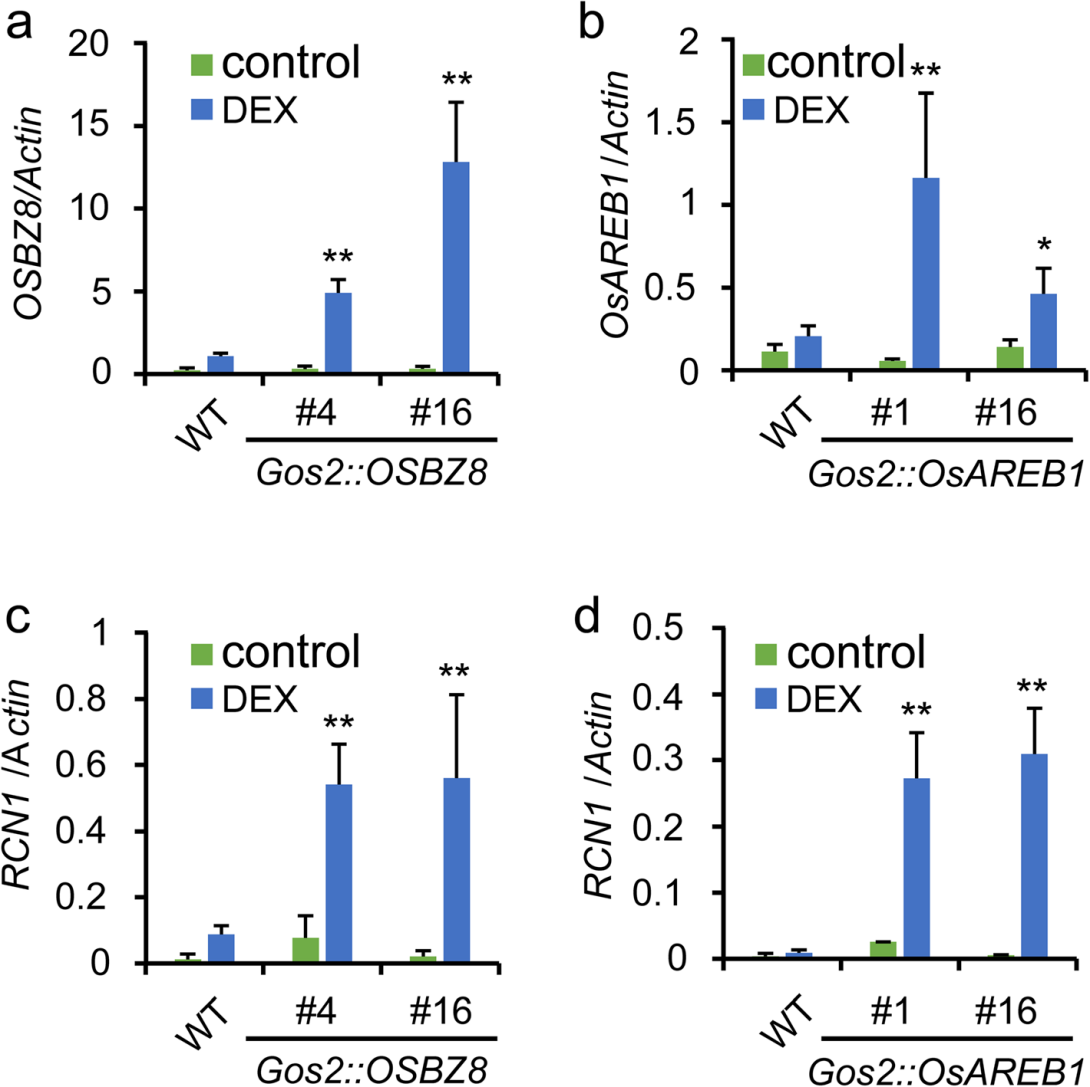
*OsAREB1-* and *OSBZ8*-induced *RCN1* expression. The expression levels of *OSBZ8* in *Gos2::OSBZ8* (**a**) and *Gos2::OsAREB1* (**b**) transgenic plants under DEX treatment. Expression levels of *RCN1* in *Gos2::OSBZ8* (**c**) and *Gos2::OsAREB1* (**d**) transgenic plants under DEX treatment. DEX: dexamethasone

The EMSA experiments revealed that OsAREB1 and OSBZ8 can bind to the 3′ distal region of the *RCN1* locus in vitro. To determine whether they can regulate *RCN1* expression in vivo, glucocorticoid-inducible transgenic lines carrying plasmid *Gos2::OsAREB1* or *Gos2::OSBZ8* in Nipponbare background were generated. The expression of *OsAREB1* and *OSBZ8* in the *Gos2::OsAREB1* and *Gos2::OSBZ8* transgenic plant leaves, respectively, was induced by a low concentration of dexamethasone (DEX) (Fig. 4a, b). As expected, the expression of *RCN1* was upregulated with the increase in *OsAREB1* and *OSBZ8* expression (Fig. 4c, d). Taken together, these findings suggest that *OsAREB1* and *OSBZ8* function as transcriptional regulators that modulate the drought response of *RCN1* via an ABA-dependent pathway.

**Fig. 5:**
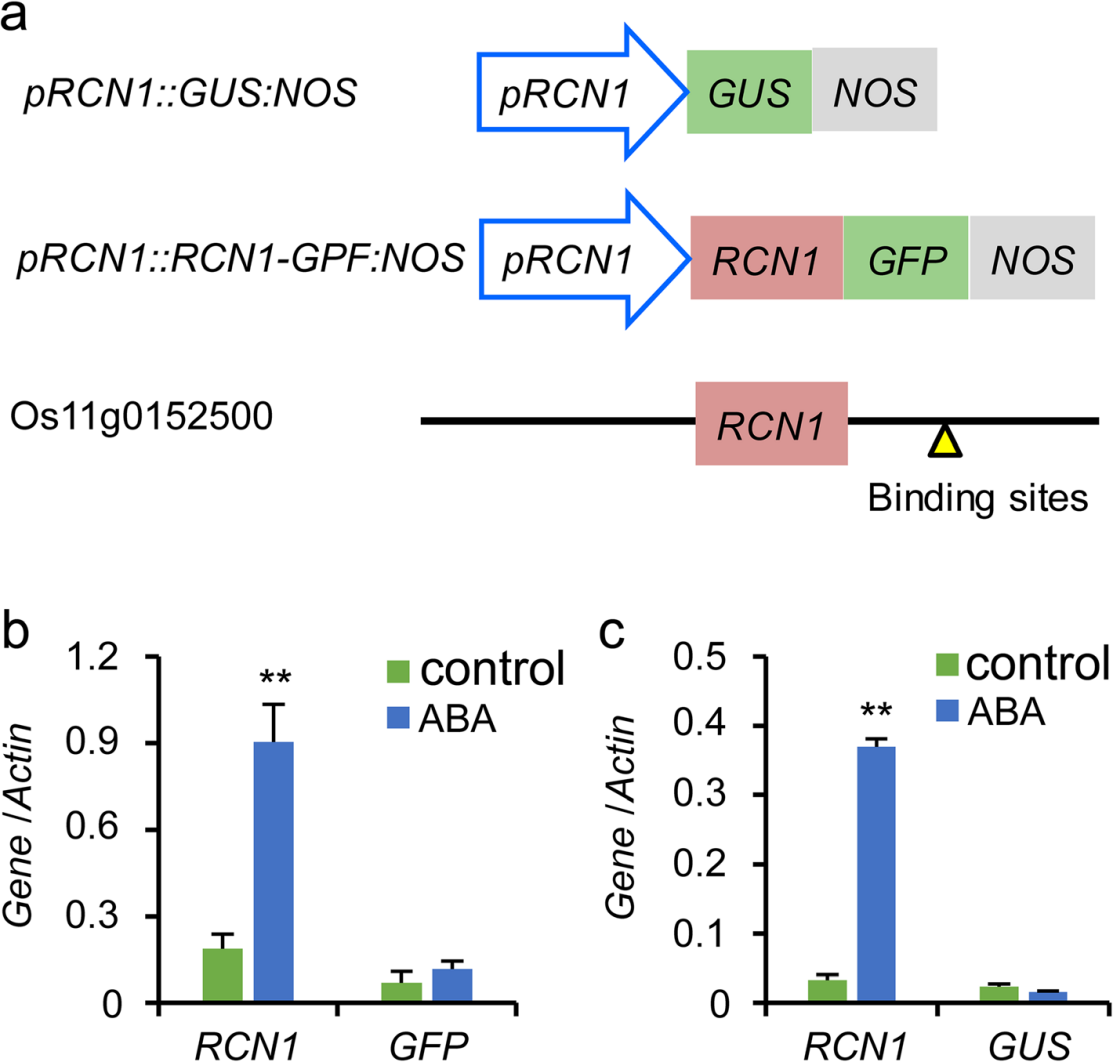
Differences in promoters and terminators in *pRCN1::GUS:NOS* and *pRCN1::RCN1-GFP:NOS* transgenic plants and wild-type (WT) plants (**a**). The expression levels of native *RCN1* and *GUS* in the *pRCN1::GUS:NOS* transgenic plants under ABA treatment (**b**). The expression levels of native *RCN1* and *GFP* in the *pRCN1::RCN1-GFP:NOS* transgenic plants under ABA treatment (**c**)

To further examine whether the 5′ proximal region of the *RCN1* locus response to ABA, we treated *pRCN1::RCN1-GFP:NOS* and *pRCN1::GUS:NOS* transgenic plants with ABA, and detected the expression levels of *GUS* and *GFP* and the endogenous expression level of *RCN1* (Fig. 5). The expression of *GFP* and *GUS* was not induced by ABA, whereas the endogenous expression of *RCN1* was significantly induced (Fig. 4b, c). The results of the qRT-PCR indicated that the ABA response element was not located on the 5′ proximal region of *RCN1*, but may be the ABRE elements on 3′ distal region of *RCN1*.

## Discussion

### *RCN1* Mediates Drought-Delayed Heading

There are two possible drought stress response modes in rice: low drought stress in the early growth stage of rice triggers the DE response (Du et al. 2018) and severe drought stress delays flowering (Bocco et al. 2012; Fukai et al. 1999; Galbiati et al. 2016; Zhang et al. 2006). In *Arabidopsis*, drought stress promoted early flowering only under LD conditions (Riboni et al. 2013; Riboni et al. 2016). However, in rice, the heading time is affected by drought stress under both SD and LD conditions (Galbiati et al. 2016), although rice is a short-day plant. In the present study, when rice was subject to drought stress, the heading of WT plants was postponed compared with that of the normally watered controls. These data agree with previous experimental results, that is, the flowering-delayed phenotype was largely observed under drought stress. In addition, the *rcn1* mutants displayed apparently earlier heading than the WT plants, implying a negative function for *RCN1* in floral transition. The heading delay of the *rcn1* mutants induced by drought was weaker than that of WT plants, suggesting that the effect of drought on the heading date of the *rcn1* mutants was less pronounced. Previous studies have identified *RCN1* as a negative floral gene, restraining floral transition by competing with Hd3a/RFT1 for 14–3-3 binding (Kaneko-Suzuki et al. 2018; Nakagawa et al. 2002; Zhang et al. 2005). A significant upregulation of *RCN1* expression was observed under drought stress; thus, drought may inhibit Hd3a protein function by promoting the expression of *RCN1*. Previous studies suggested that the delayed flowering phenotype under drought stress was largely dependent on the downregulation of *Hd3a* (Galbiati et al. 2016). Both transcriptional regulation and protein function regulation contribute to the floral transition regulatory mechanism of drought.

### Drought Induces *RCN1* in an ABA-Dependent Manner

In the present study, we showed that drought regulates *RCN1* expression through an ABA-dependent pathway. ABA is regarded as a signal that can transmit drought information when plants suffer drought stress. Under the ABA treatment, *RCN1* expression showed faster and longer-lasting upregulation in the roots than in the leaves. The root is the first organ to perceive drought stress in the plant growth process, and ABA is then rapidly synthesized and transported upward to the leaf to transmit drought information. The sensitivity of *RCN1* in the root to drought stress implies that it may play important roles in rice drought response and growth regulation. One of the ABA-mediated responses is the induced expression of a large number of genes, which are mediated by *cis*-regulatory elements, the *ABREs*. The presence of *ABRE* is essential for abiotic stress inducibility of an ABA-dependent pathway, and the transcription factor that binds to *ABRE* plays an important role in the expression of most ABA-response genes (Nakashima and Yamaguchi-Shinozaki 2013). Both *OsAREB1* and *OSBZ8* act as transcriptional regulators that modulate the ABA response of *RCN1* by directly binding with the *ABRE* located in the 3′ distal region of the *RCN1* locus. Transgenic *Arabidopsis* over-expressing *OsAREB1* had higher resistance to drought and later flowering time (Jin et al. 2010). Thus, *OsAREB1* could have the same function in rice. Our experiment showed the drought increased *OsAREB1* and *OSBZ8* expression in timing of flowering (Figure S7), thereby activating *RCN1* expression, and then causing a flowering transition delay. Our study revealed that drought delayed the heading time partially by inducing *RCN1* via an ABA signaling pathway, and that *OsAREB1* and *OSBZ8* are involved in this process.

*RCN1* showed a decreased responsiveness to drought in *rcn1* plants. Previous studies found that the Hd3a and RFT1 proteins can form a transcriptional repression complexes with BINDING REPRESSOR FACTOR 1 (HBF1) and HBF2 and then feedback to affect their own transcription in the leaves (Brambilla et al. 2017). As their homeotic gene, RCN1 might could also combine with a transcription factor to form a transcriptional complex to regulate itself in leaves.

### RCN1 Protein Is Localized to the Nuclei of Leaf and Root Cells

In *Arabidopsis*, the long-distance transport modes of FT from the phloem to shoot apex have been identified (Corbesier et al. 2007; Liu et al. 2012). The *FT*-homologous genes *Hd3a* and *RFT1* were strongly expressed in the leaves; these proteins moved from the leaf to the SAM, thereby inducing flowering (Tamaki et al. 2007; Taoka et al. 2011). As the antagonistic gene of florigen, *RCN1* was expressed in the root, leaf, and stem of rice. In the rice leaf, *RCN1* showed similar expression patterns to florigen, in the vascular bundles. The RCN1 protein was transported from the vascular tissue to the SAM (Kaneko-Suzuki et al. 2018). In the present study, *RCN1* presented the highest expression in rice roots from the vegetative to late reproductive phases. The expression of *RCN1* was relatively lower in the leaves and stems than in the roots. These results implied that *RCN1* might have important functions in the root. In the present study, *RCN1* was undetectable in the young panicle and the *RCN1* promoter activity in *gRCN-GUS* plants was also undetected in the young panicle. This might be because *RCN1* is expressed in the vascular bundle, which is not differentiated in the young panicle. Kaneko-Suzuki (2018) demonstrated the same result. We observed that the RCN1-GFP fusion protein was localized to the cytomembrane, cytoplasm, and nucleus. A previous study reported the localization of RFT1 in whole cells (Song et al. 2017) and Hd3a in the cytoplasm and nucleus (Taoka et al. 2011). The nuclear localization of *RCN1*, *Hd3a*, and *RFT1* was related to their transcription repression/activation functions that were achieved in combination with the transcription factor. Our results suggest that *RCN1* is synthesized outside the SAM in the flowering transition period to regulate flowering transition and branch differentiation. As a movement protein, RCN1 may have a signal transmission function, to respond to soil environment changes to regulate the symplastic growth of aboveground and underground plant sections.

In summary, our findings revealed that *RCN1* integrates photoperiodic and drought stress information to delay flowering transition in rice. However, the mechanism of RCN1 action is still unclear, and therefore, requires further detailed investigations.

## Materials and Methods

### Vector Construction

We used the *O. sativa japonica* cultivar (cv.) Nipponbare as the genetic background to generate the transgenic rice plants. To examine the expression pattern of *RCN1*, its promoter (4 kb upstream of ATG) was first amplified by PCR using the genomic DNA of rice, and then inserted into the pCAMIBA1300 vector, which contained the GUS reporter group and the NOS terminator, to generate the *pRCN1::GUS:NOS* construct. A similar method was used to generate the construct containing GFP. The full length *RCN1* coding region was amplified from rice by PCR, and then cloned into the region between the *RCN1* promoter and GFP in *pRCN1::GUS:NOS* to obtain the *pRCN1::RCN1-GFP:NOS* construct. To generate the DEX-inducible overexpression construct of *OsAREB1* and *OSBZ8*, we amplified the full-length cDNAs of *OsAREB1* and *OSBZ8* from the genomic DNA by PCR. Fragments of *OsAREB1* and *OSBZ8* were inserted into the pINDEX vector containing the *Gos2* promoter, which can be induced by low concentrations (1–10 μM) of DEX (Ouwerkerk et al. 2001). The CRISPR-Cas9 procedure was performed as previously described (Mao et al. 2013). Two different single-guide RNA oligos targeting *RCN1* were designed based on the first and second exons and transformed into Nipponbare. The *rcn1–4* and *rcn1–11* mutants were used in this study (Figure S1). The primers used in vector construction are shown in Table S1.

### Plant Growth Conditions and Drought Stress Experiment

The wild-type and *rcn1* mutant plants (two weeks after germination) were grown during the growing season. Flowering time was measured as the number of days from germination to heading. For the drought stress experiments, the plants were grown on nutrient soil in cylindrical boxes (diameter = 20 cm, height = 21 cm). After two weeks, half of the plants were subject to drought stress. Each cylindrical box was filled with nutrient soil and air-dried for 72 h in an oven at 75 °C and weighed to determine dry weight. The cylindrical boxes were then immersed in water for 24 h and the wet weight was determined. The soil water capacity (SWC) was calculated as the difference between wet weight (full water) and dry weight. The soil relative water content (SRWC) was calculated as: (current wet weight - dry weight) / SWC × 100. When rice leaves started to curl, the SRWC was 50%. Fifteen plants were tested for each genotype in both control and drought-treated groups. The cylindrical boxes were weighed every day; nutrient solution was added to maintain the SRWC at 30%–50% (severe drought stress). Sufficient water was supplied to the control group, and both treatment and control groups were supplied with sufficient nutrient solution. The first fully expanded leaf was sampled in at 50 DAS for transcript level analysis under normally watered/drought stress conditions.

### Plant Culture and Treatments

Rice cv. Wuyujing3 seeds were sown individually in 96-well plates. The plates were then floated on rectangular plastic trays (32.5 cm × 21.5 cm × 11.5 cm) filled with 4 L of modified Yoshida nutrient solution containing 1.0 mM NH_4_NO_3_, 1.0 mM CaCl_2_, 1.7 mM MgSO_4_, 0.5 mM K_2_SO_4_, 320.0 μM NaH_2_PO_4_, 36.0 μM Fe (III)-EDTA, 18.0 μM H_3_BO_3_, 9.1 μM MnCl_2_, 0.15 μM ZnSO_4_, 0.16 μM CuSO_4_, 0.074 μM (NH_4_)_6_Mo_7_O_24_, and 40.5 μM citric acid (Ding et al. 2018). The nutrient solution was refreshed every five days to maintain a pH of 5.5. The plants were grown in climate chambers under LD conditions (14 h light at 28 °C /10 h dark at 24 °C) with a relative humidity of ~ 70%, and treated for 14 days.

The effect of water stress on *RCN1* expression was studied using PEG6000 dissolved in nutrient solution at 10%, 20%, or 30% concentration. Plants grown in the nutrient solution without PEG6000 were used as the controls. Root and shoot samples were collected after 24 h of treatment and frozen in liquid nitrogen at − 80 °C.

Plants were treated with ABA at different concentrations and for different times. The stock solution of ABA was dissolved in ethanol and diluted in modified Yoshida nutrient solution. Rice plants were treated with 10 μM ABA; root and shoot samples were collected after 0 (control), 3, 6, 24, 30, 48, 54, 96, and 102 h, and frozen in liquid nitrogen at − 80 °C. Five concentrations of ABA (0.4, 2, 10, 50, and 250 μM) were used, plus a control treatment with an equal amount of the solvent. Root and shoot samples were collected after 24 h of ABA treatment and frozen in liquid nitrogen at − 80 °C.

As the main hormone of the drought response pathway, ABA may play a key role in regulating the response of *RCN1* to drought. To verify this hypothesis, further experiments were conducted to identify whether PEG6000 induces *RCN1* expression in an ABA-dependent manner. 48 rice plants were treated with fluridone (40 μΜ) for 30 min, and then moved into 30% PEG6000 solution containing fluridone. Thirty rice plants were also treated with 50 mM CHX for 30 min to block protein synthesis, and 30 plants did not receive the treatment. The CHX-treated and untreated rice plants were then divided into two groups, each treated with the nutrient solution or 50 μM ABA. In the ABA-signaling pathway, the released SNF1-related *protein* kinase 2 activates downstream transcription factors by protein phosphorylation, and then promotes the expression of ABA-response genes (Kerr et al. 2018). Thus, 60 rice plants were pretreated with K252a (50 nM), which is a phosphorylation blocker, while 60 plants did not receive the treatment. The K252a-treated and untreated rice plants were then divided into two groups, and each of these plants was further treated with 30% PEG6000 or 50 μM ABA.

Twenty plants each of *Gos2::OsAREB1*, *Gos2::OSBZ8*, and Nipponbare lines, were treated with DEX powder dissolved in ethanol and diluted to 10 μΜ in modified Yoshida nutrient solution for 24 h, while 20 plants were treated with an equal amount of the solvent and used as the controls. Forty plants each of *pRCN1::GUS:NOS* and *pRCN1::RCN1-GFP:NOS* lines were grown in water culture under LD condition (14 h light at 28 °C /10 h dark at 24 °C). Half of the plants were treated with 50 μM ABA for 6 h, while the other half was left untreated and used as the controls.

### RNA Extraction and qRT-PCR Analysis

The total RNA was extracted from rice tissues with using the E.Z.N.A.® Total RNA Kit I (Omega Bio-tek, Norcross, GA, USA) following the manufacturer’s instructions. The total RNA samples were treated with RQ1 DNase (Promega, Madison, WI, USA) at 37 °C for 30 min to remove genomic DNA, and then converted to cDNA with iScript™ Reverse Transcription Supermix (Bio-Rad, Hercules, CA, USA). A qRT-PCR was conducted in the CFX Connect Real Time System (Bio-Rad) using the iTaq Universal SYBR Green Supermix (Bio-Rad). The thermal cycle conditions were as follows: 95 °C for 3 min and 40 cycles at 95 °C for 15 s and 60 °C for 60 s, followed by melting curve analysis to verify the specificity of amplification. The ΔΔCt method was used to calculate relative expression levels using the rice *Actin* gene as the internal control (Arocho et al. 2006). The primers used in the qRT-PCR are shown in Table S2. The values presented are the mean of three biological replicates, each of which had three technical replicates.

### Histochemical Analysis of GUS and Fluorescence Imaging

GUS staining was performed as previously reported (Tamaki et al. 2007). The GFP fluorescence was detected using the LSM710 META confocal laser-scanning microscope (Carl Zeiss AG, Oberkochen, Germany) as previously reported (Tamaki et al. 2007).

### Transient Expression of Proteins in *Nicotiana benthamiana* Leaf Epidermal Cells

The *35S::RCN1-GFP::NOS* vector was acquired from BioRun (Shanghai, P. R. China) and the full length *RCN1* coding region was inserted into the vector that contained the *GFP* reporter group and 35S promoter. The vector was transformed into tobacco (*N. benthamiana*) leaves using *Agrobacterium tumefaciens* strain *EHA105* (Lee et al. 2005). The GFP fluorescence was observed under a confocal laser microscope (LSM710) after preparation of microscope slides with tobacco leaves at 5 days post-infection.

### Protein Expression

The *OSBZ8*, *OsAREB1*, and *TRAB1* coding regions were cloned into the pCold™ vector (Code No. 3361; Takara Bio Inc., Kusatsu, Japan) and the corresponding proteins were expressed in *Escherichia coli* BL21 strain according to the manufacturer’s instructions. Collected cells were resuspended in phosphate buffer saline (NaCl 137 mM, KCl 2.7 mM, Na_2_HPO_4_ 10 mM, KH_2_PO_4_ 2 mM; pH = 7.2) containing the cOmplete™ Protease Inhibitor Cocktail (Roche, Basel, Switzerland) and lysed with ultrasound. The suspension was centrifuged at 4000×g for 10 min at 4 °C, and the resulting supernatant was used for further experiments. The lysates were purified using the Capturem™ His-Tagged Purification Miniprep Kit (Takara Bio Inc., Kusatsu, Japan) according to the manufacturer’s instructions.

### EMSA

The EMSA experiments were performed on the 7-kb upstream and 2-kb downstream DNA sequences of *RCN1*. Eleven DNA fragments containing 19 ACGT were synthesized and labeled with biotin (Table S3). The DNA-binding reaction was performed using the Chemiluminescent Nucleic Acid Detection Module Kit (Thermo Fisher Scientific, Waltham, MA, USA) following the manufacturer’s instructions.

### Statistical Analyses

The results are presented as mean ± standard error. The data were analyzed using a two-tailed paired Student’s *t* test.

## Supplementary information

**Additional file 1: Figure S1.** Mutational sites of *RCN1* in *rcn1–4* and *rcn1–11* mutants. **Figure S2.** Schematic diagram of the experimental timetable for drought treatment. SRWC: soil relative water content. **Figure S3.** The expression levels of *RCN1* (a), *Hd3a* (b), and *RFT1* (c) in the leaves response to drought. **Figure S4.** Localization of the RCN1-GFP protein in tobacco leaf epidermal cells (a) and rice protoplasts (b). **Figure S5.** Expression levels of *bZIP* family transcription factors under ABA treatment in rice roots, according to the RiceXpro data (a). Expression levels of *OsAREB1*, *TRAB1*, *OSBZ8*, *RITA*, *bZIP23*, and *RCN1* in rice roots (b) and leaves (c) under ABA treatment. Expression levels of *OsAREB1*, *OSBZ8*, and *TRAB1* in rice roots (d) and leaves (e) after CHX and ABA treatments. CHX: cycloheximide, a type of protein de novo synthesis blocker. **Figure S6.** Gel electrophoresis revealed that OsAREB1 (a) and OSBZ8 (b) were not bound to the upstream sequence of *RCN1*; TRAB1 was not bound to the sequence of *RCN1* (c). **Figure S7.** The expression levels of *OsAREB1* (a), *OSBZ8* (b) in the leaves response to drought at the timing of flowering. **Table S1.** Primers used for vector construction. **Table S2.** Primers used for qRT-PCR. **Table S3.** Probes used for EMSA experiment.

## Data Availability

The datasets supporting the conclusions of this article are included within the article and its additional files.

## Abbreviations

ABA: Abscisic acid
CHX: Cycloheximide
CRISPR-Cas9: Clustered regularly interspaced short palindromic repeats-associated protein-9
DE: Drought-escape
DEX: Dexamethasone
EMSA: Electrophoretic mobility shift assays
GFP: GREEN FLUORESCENT PROTEIN
GUS: Beta-glucuronidase
LD: Long-day
PEG6000: Polyethylene glycol
qRT-PCR: Quantitative reverse transcription- PCR
SAM: Shoot apical meristem
SRWC: Soil relative water content
SWC: Soil water capacity
WT: Wild type
