# Supplementary material for: RICE CENTRORADIALIS 1, a TFL1-like Gene, Responses to Drought Stress and Regulates Rice Flowering Transition
Source: Rice (N Y). 2020 Sep 24;13:70. doi: 10.1186/s12284-020-00430-3 (PMC7516004; doi:10.1186/s12284-020-00430-3)
Supplement: Supplementary file 1 — Additional file 1: Figure S1. Mutational sites of RCN1 in rcn1–4 and rcn1–11 mutants. Figure S2. Schematic diagram of the experimental timetable for drought treatment. SRWC: soil relative water content. Figure S3. The expression levels of RCN1 (a), Hd3a (b), and RFT1 (c) in the leaves response to drought. Figure S4. Localization of the RCN1-GFP protein in tobacco leaf epidermal cells (a) and rice protoplasts (b). Figure S5. Expression levels of bZIP family transcription factors under ABA treatment in rice roots, according to the RiceXpro data (a). Expression levels of OsAREB1, TRAB1, OSBZ8, RITA, bZIP23, and RCN1 in rice roots (b) and leaves (c) under ABA treatment. Expression levels of OsAREB1, OSBZ8, and TRAB1 in rice roots (d) and leaves (e) after CHX and ABA treatments. CHX: cycloheximide, a type of protein de novo synthesis blocker. Figure S6. Gel electrophoresis revealed that OsAREB1 (a) and OSBZ8 (b) were not bound to the upstream sequence of RCN1; TRAB1 was not bound to the sequence of RCN1 (c). Figure S7. The expression levels of OsAREB1 (a), OSBZ8 (b) in the leaves response to drought at the timing of flowering. Table S1. Primers used for vector construction. Table S2. Primers used for qRT-PCR. Table S3. Probes used for EMSA experiment. [file 12284_2020_430_MOESM1_ESM.docx]

**
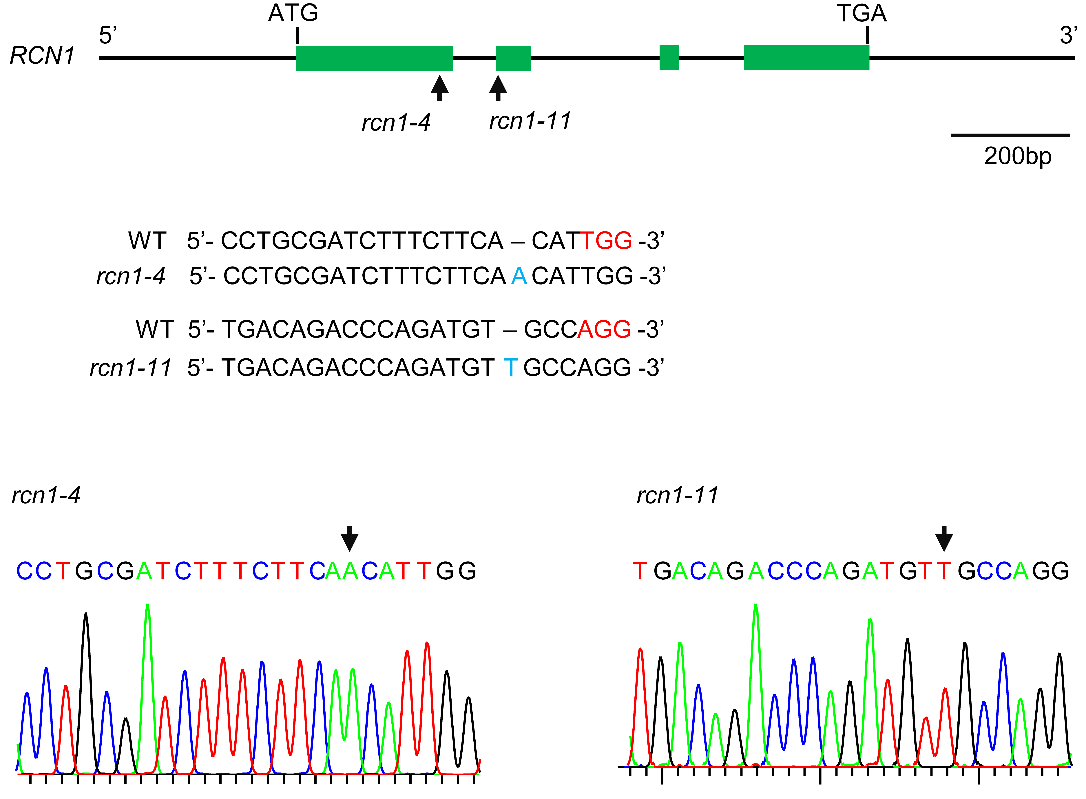
**

**Figure S1.** Mutational sites of *RCN1* in *rcn1-4* and *rcn1-11* mutants

**
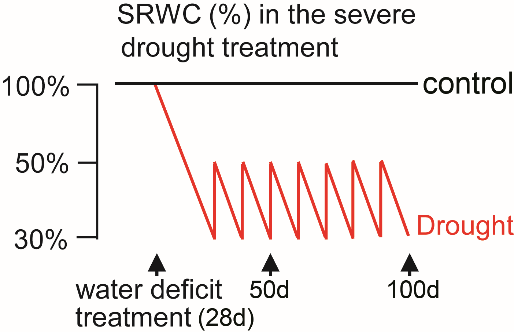
**

**Figure S2.** Schematic diagram of the experimental timetable for drought treatment. SRWC: soil relative water content.


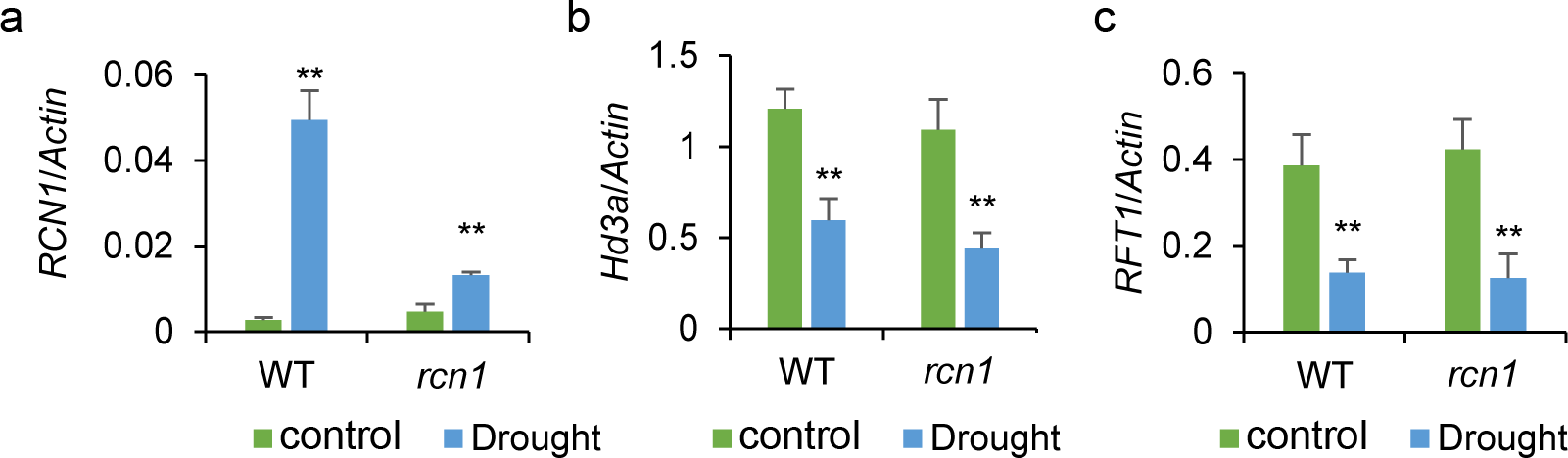


**Figure S3.** The expression levels of *RCN1* (a), *Hd3a* (b), and *RFT1* (c) in the leaves response to drought.

**
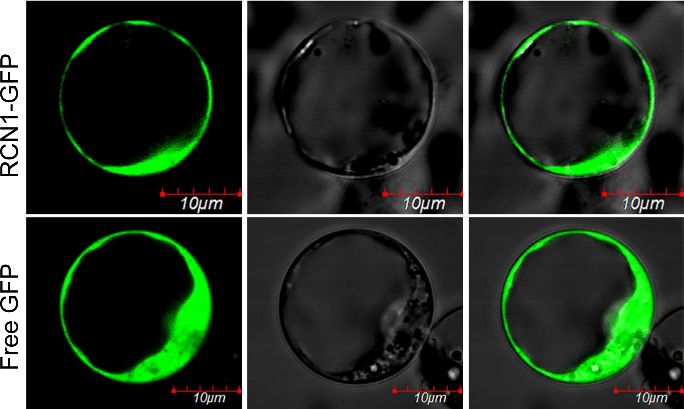
**

**Figure S4.** Localization of the RCN1-GFP protein in rice protoplasts.

**
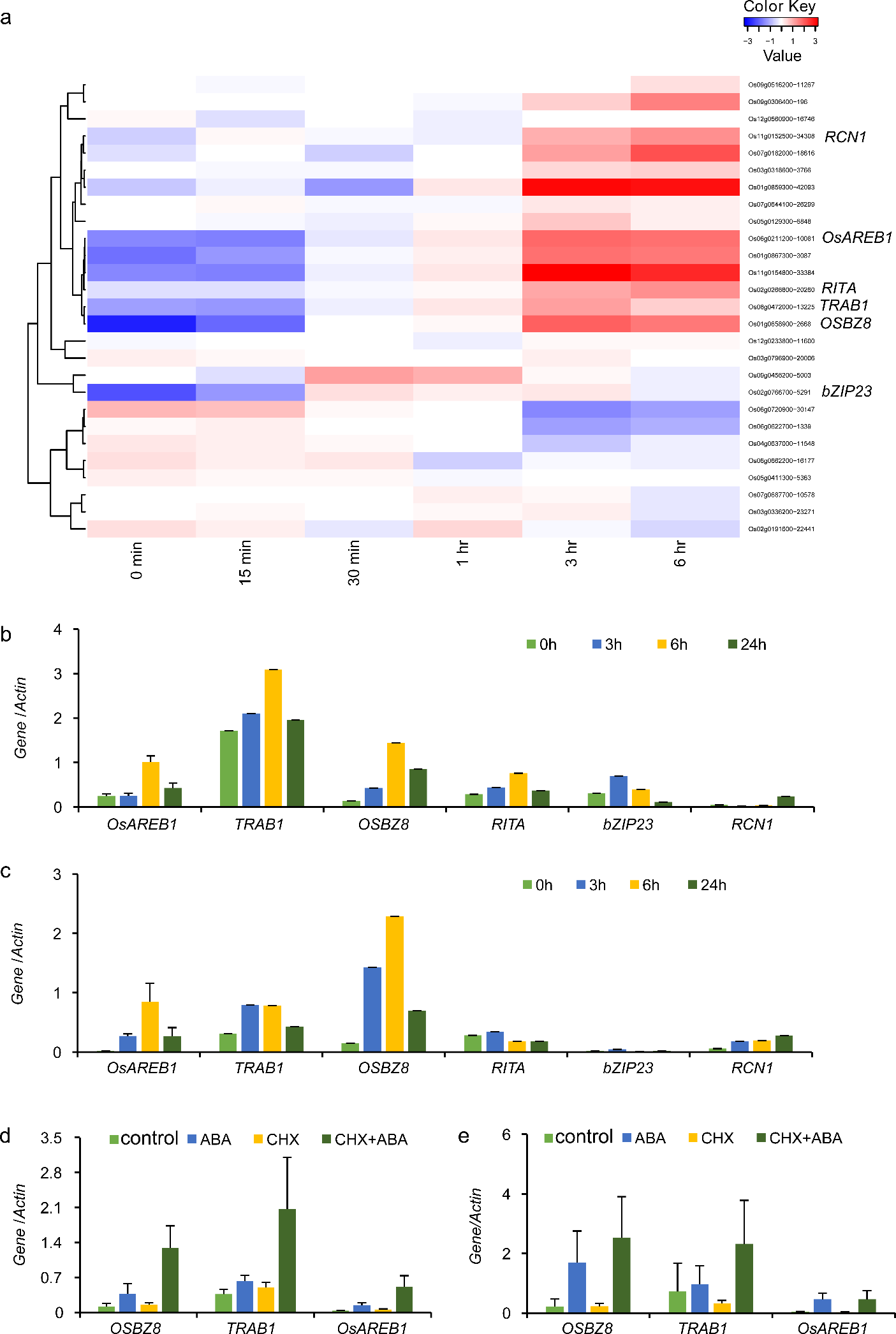
**

**Figure S5.** Expression levels of *bZIP* family transcription factors under ABA treatment in rice roots, according to the RiceXpro data (a). Expression levels of *OsAREB1*, *TRAB1*, *OSBZ8*, *RITA*, *bZIP23*, and *RCN1* in rice roots (b) and leaves (c) under ABA treatment. Expression levels of *OsAREB1*, *OSBZ8*, and *TRAB1* in rice roots (d) and leaves (e) after CHX and ABA treatments. CHX: cycloheximide, a type of protein *de novo* synthesis blocker.

**
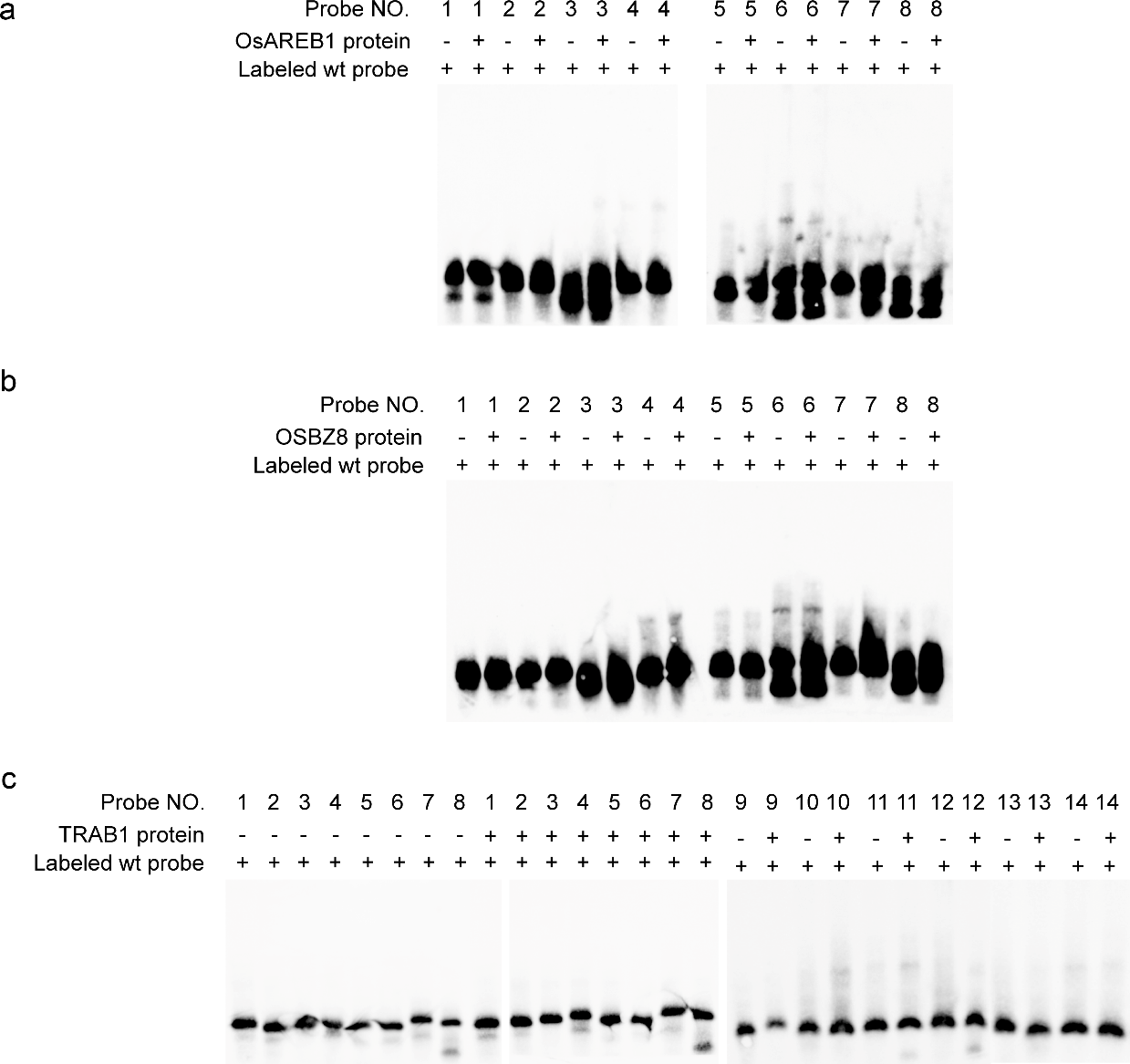
**

**Figure S6.** Gel electrophoresis revealed that OsAREB1 (a) and OSBZ8 (b) were not bound to the upstream sequence of *RCN1*; TRAB1 was not bound to the sequence of *RCN1* (c).


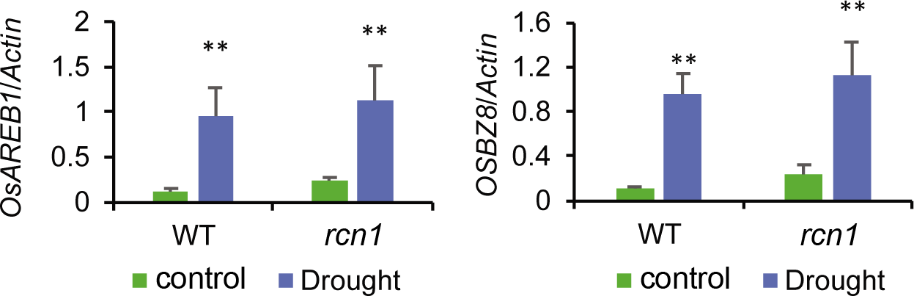


Figure S7. The expression levels of OsAREB1 (a), OSBZ8 (b), in the leaves response to drought at the timing of flowering.

**Table S1.** Primers used for vector construction.

| Vector Name | Primers | Forward primer 5’-3’ | Reverse primer 5’-3’ |
| --- | --- | --- | --- |
| *pRCN1::RCN1-GFP:NOS* | *RCN1-mRNA* | TAAGGATCCACAGCTCACCGAAGTGC | AATGGTACCGCGCCTCCTGGCTGCAG |
|  | *GFP-NOS* | CTAGGTACCATGGTGAGCAAGGGCGAGG | CGATGAATTCGATCTAGTAACATAGATG |
| *pRCN1::GUS:NOS* | *RCN1-promoter* | GTCGACTTGCGAGACATTTCAGCAGCAA | CTGTGGATCCGGGCTGATTGCTTGGCAATG |
| *Gos2::OsAREB1* | *OsAREB1-CDS* | CATATGGAGTTGCCGGCGGA | GAATTCTTACCATGGACCAG |
| *Gos2:: OSBZ8* | *OSBZ8-CDS* | CATATGGGAAATGACGAAGCTG | GAATTCTTACCTTGCGGCTACAGCA |

**Table S2.** Primers used for qRT-PCR.

| Gene | Forward primer 5’-3’ | Reverse primer 5’-3’ |
| --- | --- | --- |
| *Actin* | CAATCGTGAGAAGATGACCC | GTCCATCAGGAAGCTCGTAGC |
| *bZIP23* | ATGGCTTCGGCAAGATGGAA | GTTGGCCTACAAGGGCAGAT |
| *GFP* | ACCATGGTGAGCAAGGGCGAGG | AGCTCGTCCATGCCGTGAGTGAT |
| *GUS* | CTTTACTGGCTTTGGTCGTC | CAATACTCCACATCACCACG |
| *Hd3a* | CAGCGTCATTTGGGCAAGAG | AGTGAGCATGCAGCAGATCG |
| *OsAREB1* | TGCCTTACCCATTCGACACC | CATGGACCAGTCAGTGT |
| *OSBZ8* | CAGGCTAACGGAAAGCTCCA | TACAGCATCAGTCGCCAGAC |
| *RCN1* | ACTGTCTGCTCCTCTAAACA | GGCTGCTGATGTAGCAGAAG |
| *RFT1* | TTAATTTGACATGGCCGGCAG | GCCCAAATGTTGCTCCAGTG |
| *RITA* | ATGGTTTCCAACCGGGAGTC | CAGGACGACACTTGACACGA |
| *TRAB1* | ACGGACAAAAGAAGCGTTGC | GAGGGGGACATAAGAGCACG |

**Table S3.** Probes used for EMSA experiment.

| Probe NO. | Sequence | Numbers of *AREB* element |
| --- | --- | --- |
| 1 | ATCGTGTTCTTCGCCGGC**ACGT**GGTAATGGGCAGAATCTCC | 1 |
| 2 | AGCTTTGAAACATTTTCC**ACGT**TTCATGAACCAACACTCAAT | 1 |
| 3 | GTCATTTTGAGGGGCTG**ACGT**TTGGTGGACAGCGCAGTTT | 1 |
| 4 | TTGAGTTTTTGCTTGCA**ACGT**TTGACCACTCGTCTTATTC | 1 |
| 5 | ATAAGACAAGTGGTCAA**ACGT**TGCAAACAAAAACTCAAAA | 1 |
| 6 | TGTGGGACGGAGGGAGT**ACGT**CACAAAAATTATGTTACAA | 1 |
| 7 | TCCTACATACCCTCAGC**ACGT**AAAGCGAGAAGTTTTATCT | 1 |
| 8 | GATAAGATTGCTCAGCA**ACGT**AATCTTCATAGATCCACTT | 1 |
| 9 | TGTGGTTTTGGCTG**ACGT**GGCGCCT**ACGT**GGCGAATTTGACTC | 2 |
| 10 | TCTTCATCTG**ACGT**GGCATTGATGTGGTGCTT**ACGT**GGCACTTCGA | 2 |
| 11 | AATAAAAATGGTGGGACCC**ACGT**GGGCCCCACAAGTCATTCT | 1 |
| 12 | AGTCAAATTAGCC**ACGT**AGGCGAC**ACGT**CAGCTAAAACCGC | 2 |
| 13 | CTTCATCTG**ACGT**GACACTG**ACGT**GGCGCTT**ACGT**GGCAAGTTGA | 3 |
| 14 | AGGAAAAATGGTGGGACCC**ACGT**GGGCCCCACATGTCATCCT | 1 |
| Mutant-9 | TGTGGTTTTGGCTGGGCGCCTGGCGAATTTGACTC | 0 |
| Mutant-10 | TCTTCATCTGGGCATTGATGTGGTGCTTGGCACTTCGA | 0 |
| Mutant-11 | AATAAAAATGGTGGGACCCGGGCCCCACAAGTCATTCT | 0 |
| Mutant-12 | AGTCAAATTAGCCAGGCGACCAGCTAAAACCGC | 0 |
| Mutant-13 | CTTCATCTGGACACTGGGCGCTTGGCAAGTTGA | 0 |
| Mutant-14 | AGGAAAAATGGTGGGACCCGGGCCCCACATGTCATCCT | 0 |
